# Nanocomposite structure of two-line ferrihydrite powder from total scattering

**DOI:** 10.1038/s42004-020-0269-2

**Published:** 2020-02-21

**Authors:** Nicholas P. Funnell, Maxwell F. Fulford, Sayako Inoué, Karel Kletetschka, F. Marc Michel, Andrew L. Goodwin

**Affiliations:** 1 Department of Chemistry, University of Oxford, Inorganic Chemistry Laboratory, South Parks Road, Oxford, OX1 3QR UK; 2 ISIS Neutron and Muon Facility, Rutherford Appleton Laboratory, Didcot, OX11 0QX UK; 3 Department of Physics, Kings College London, Strand, London, WC2R 2LS UK; 4 Department of Geosciences, Virginia Tech, Blacksburg, VA 24061 USA

**Keywords:** Atomistic models, Nanoparticles, Geochemistry, Characterization and analytical techniques

## Abstract

Ferrihydrite is one of the most important iron-containing minerals on Earth. Yet determination of its atomic-scale structure has been frustrated by its intrinsically poor crystallinity. The key difficulty is that physically-different models can appear consistent with the same experimental data. Using X-ray total scattering and a nancomposite reverse Monte Carlo approach, we evaluate the two principal contending models—one a multi-phase system without tetrahedral iron(III), and the other a single phase with tetrahedral iron(III). Our methodology is unique in considering explicitly the complex nanocomposite structure the material adopts: namely, crystalline domains embedded in a poorly-ordered matrix. The multi-phase model requires unphysical structural rearrangements to fit the data, whereas the single-phase model accounts for the data straightforwardly. Hence the latter provides the more accurate description of the short- and intermediate-range order of ferrihydrite. We discuss how this approach might allow experiment-driven (in)validation of complex models for important nanostructured phases beyond ferrihydrite.

## Abstract (web summary)

Although a geologically important mineral, the atomic-scale structure of ferrihydrite remains unresolved. Here the authors combine X-ray total scattering and reverse Monto Carlo to evaluate the two principal contending models, explicitly considering the material’s complex nanocomposite structure.

## Introduction

Ferrihydrite is a nanoparticulate ferric oxyhydroxide of unique importance across a range of fields^1^: it is widespread in soils and freshwater environments^2,3^; it forms a core structural component in the Fe-buffering ferritin protein, found in numerous organisms^4,5^; it is a major component of Fe-containing minerals on Mars^6^; and it is used in the sequestration of heavy element contaminants^7^. Structurally, it is known to be a precursor of haematite and goethite^8^, yet its own three-dimensional atomic structure has remained the subject of intense debate^9–14^. The key difficulties in determining its structure are a combination of a very small particle diameter—giving rise to severe powder diffraction peak broadening—and a high degree of defect incorporation.

**Fig. 1:**
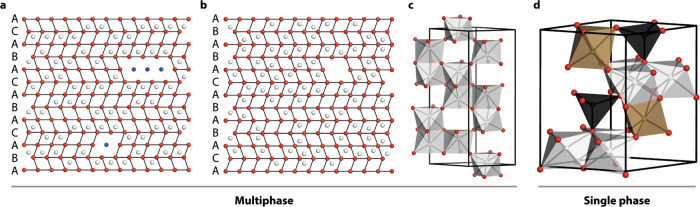
Candidate ferrihydrite models. **a** Representative slices of non-defective and **b** defective particles in the multiphase (MP) model, viewed along the 〈110〉 direction. White spheres represent Fe atoms, red, O atoms, and blue, water molecules. Both figures are shown in the same style as Fig. 1a in ref. ^9^ to aid comparison. O anion vacancies can occur in defective particles where all neighbouring Fe atoms are randomly vacant—inclusion of oxygen would be equivalent to the presence of water. **c** Haematite structure, which forms a third, minority, component of the MP model. **d** Single-phase (SP) model, where Fe1, Fe2 and Fe3 sites are shown as light grey, brown and black polyhedra, respectively.

In this respect, ferrihydrite is representative of an important general challenge facing structural science: namely, how might we develop robust protocols for determining the structure of nanoscale materials^15–17^? The conventional—and most straightforward—approach is to approximate nanomaterials as periodic crystals modified by a suitable shape function. This interpretation is implicit in Debye–Sherrer analysis of diffraction peak widths but is also sometimes carried out explicitly via modelling of the pair distribution function (PDF) with appropriate damping functions^18,19^. This is precisely the approach that has been applied to the problem of ferrihydrite, such that from amongst the variety of structural models proposed over the last fifty years^20–22^, essentially only two competing models remain as the basis for ongoing debate within the community^10,23^. One is the multi-phase model proposed by Drits et al.^23^ that contains a mixture of defect-free and defective ferrihydrite nanocrystals together with ‘ultra-dispersed’ haematite (Fig. 1a–c); we refer to this as the ‘MP’ model. The second is the single-phase akdalaite-like model of Michel et al.^10,24^ (Fig. 1d); we refer to this as the ‘SP’ model. Diffraction and PDF measurements have proven insufficiently discriminating to rule out either model in any definitive sense, although recent experimental and theoretical studies support the case for the SP structure^14,25^. The difficulty in all comparative studies is that the MP model requires significant simplifications^26^ to allow its complex composition to be approximated by a single damped periodic structure.

Implicit in the interpretation of scattering data in terms of damped periodic models is the assumption that the nanoparticle surface structure is identical to that of the core (or, equivalently, the surface constitutes a sufficiently small volume fraction that it might safely be ignored). In some instances this approximation is justified: nanoparticulate Au is probably the least controversial example^27^. However, in the case of the most disordered ferrihydrites (so-called ‘2-line’ phases) the structural coherence length is just 2–3 nm^28^, which requires that half of the atoms in each nanocrystal lie within 3 Å of the surface; significant surface relaxation is expected in such cases^29^. A cumbersome—though ultimately unavoidable—work-around is to reproduce scattering data from atomistic (‘big box’) rather than periodic (‘small box’) structural models^30^. Here the concept is to build a realistic nano-scale model of the ferrihydrite structure and then to refine atom positions within this model using an approach, such as reverse Monte Carlo (RMC) refinement^31^. To date, probably the most comprehensive atomistic reproduction of 6-line ferrihydrite is that reported in ref. ^32^, where RMC methods were used to refine atom positions within a single nanoparticle of diameter 3.6 nm—incorporating structural defects in a more realistic manner than approximations to a periodic cell can achieve. Even in this atomistic representation, the MP model was heavily simplified because its multiple constituent phases were approximated by a single nanoparticle.

In fact, experiment shows that individual nanoparticle models—whatever their complexity—will never accurately describe the nanostructure of 2-line ferrihydrite. High-resolution transmission electron microscopy (HRTEM) measurements reveal that poorly-defined nanocrystals coexist with an amorphous component (matrix) of a similar chemical composition^33^. Similar complexity is present in other important iron-containing nanomaterials, such as schwertmannite^34^ and may arise as a consequence of a complex crystallisation pathway^35^. Although the amorphous matrix of ferrihydrite may lack long-range order, its constituent atoms cannot be randomly arranged. Consequently, the matrix contributes meaningfully to the PDF at low interatomic separations, even if its signature in the diffraction pattern (i.e., in reciprocal space) is not highly structured. Moreover, the MP model includes multiple nanocrystalline components. Taken together, these considerations mean that the only method by which the SP and MP models might be compared on equal footing is by constructing even larger atomistic models that represent an entire nanocomposite assembly that explicitly includes both nanoparticle and matrix components.

In this study, we address this challenge directly by extending the RMC approach to nanocomposite heterostructures. While RMC methods have long been used to determine the structures of crystalline, amorphous, and nanocrystalline materials^36–39^, our methodology is distinct in using a single atomistic model that incorporates at once both nanocrystalline and amorphous components. We build complete atomistic representations of the SP and MP models that are as faithful as possible to the original descriptions laid out in refs. ^23,24^. Full structural details of the models are given in Supplementary Note 1 and Supplementary Tables 1–3. Our models differ from those of previous studies in that our configurations are an ensemble of several individual nanoparticles, embedded in a structured amorphous, non-random matrix. The structure of each particle follows as faithfully as possible the relevant models described in the literature, without further simplifications. A long-standing criticism of both MP and SP models has been that they do not account at once for both local and long-range features—we assess this directly by using RMC refinements to make perturbative adjustments in atomic positions, fitting simultaneously to real and reciprocal space data. We interrogate the models in terms of quality of fit-to-data, of the physicality of their pairwise correlation functions, and the extent and nature of structural reorganisation introduced in the fitting process. We will show that only the SP model provides a physical representation of the structure of 2-line ferrihydrite, and that the closest unit-cell description is a marginally less distorted variant of the akdalaite model of ref. ^24^.

## Results

### Model generation

We begin with a brief description of our approach for generating atomistic representations of ferrihydrite for subsequent RMC analysis; full details are provided in the Supplementary Methods. The nanoparticle and matrix components of the nanocomposite structure are prepared individually before being combined to give the starting RMC model. In all cases we omit H atoms, as required by the correspondingly poor sensitivity in X-ray scattering data.

For the nanoparticle component, we generate each nanoparticle starting from a conventional atomistic configuration of the corresponding phase. The MP model contains spherical nanoparticles of so-called ‘defect-free’ (f-phase, ordered stacking sequence, Fig. 1a) and ‘defective’ (d-phase, disordered stacking sequence, Fig. 1b) ferrihydrite phases together with ultra-dispersed cylindrical nanoparticles of haematite (Fig. 1c). The SP model consists of a single nanoparticle type that is isostructural with akdalaite, Al_10_O_14_(OH)_2_ (Fig. 1d)^10,24^. We note in passing that an important distinction between the phases present in these models is the absence (MP) or presence (SP) of tetrahedrally-coordinated Fe^3+^^11,40–42^. In each case a large atomistic configuration is generated, a spherical (ferrihydrite) or cylindrical (haematite) section of appropriate dimensions^23^ is excised and then surface Fe atoms of this spherical particle removed if under-coordinated. Individual nanoparticles are then positioned and oriented randomly (subject to hard-sphere constraints) within the final box; at this stage we aim for a filling ratio of  ~50%, achieved using a hard-sphere Monte Carlo (MC) algorithm. In the case of the MP configuration we also ensure that the correct volume ratio of defect-free, defective, and haematite nanoparticles has been incorporated^23^.

To prepare the matrix component, we fill a separate configuration (of the same size as the final box) with Fe and O atoms according to the bulk ferrihydrite composition and number density. These atoms are initially placed randomly, but sensible local order is introduced through a MC algorithm that is first driven purely by hard sphere constraints and subsequently by local distance restraints informed by a relevant crystalline phase. The result is a disordered configuration with sensible composition, density, and local Fe and O coordination. The matrix and multi-nanoparticle boxes are then combined, removing from the matrix component any atoms that overlap with nanoparticles.

### Ruling out the multi-phase model

**Fig. 2:**
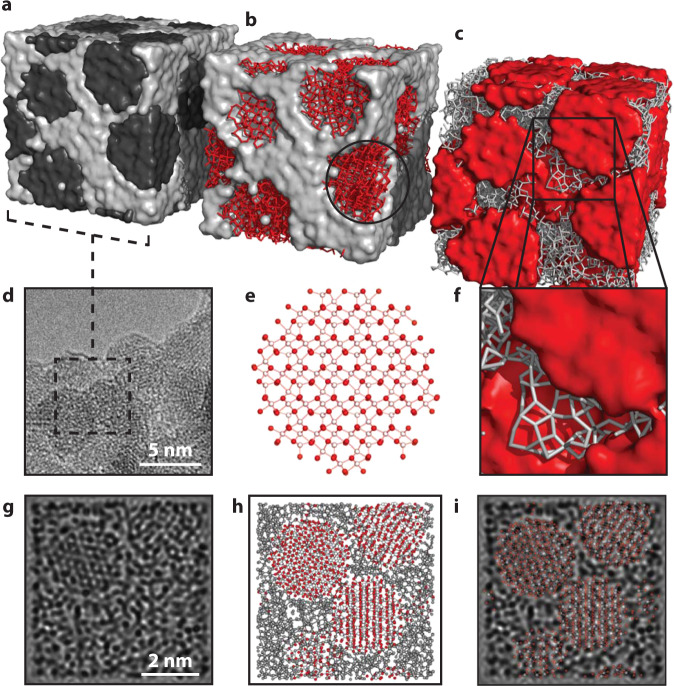
Nanocomposite models and microscopy. **a** Nanocomposite simulation box (60 Å)^3^, with particle and matrix surfaces shown in black and grey, respectively, illustrating its resemblance to the TEM image, shown in **d**—the enclosed region shows the approximate cubic volume covered by the simulation box. **b** Simulation box schematic, highlighting the structure of the particles, shown in red, and **c** the matrix, shown in grey. **e** A typical particle model, prior to refinement, representative of the circled region in (**b**). O atoms are coloured red and Fe, white. **f** Expanded region of (**c**), emphasising the reasonable atomic connectivity in the matrix structure. **g** Simulated TEM micrograph, calculated from a 10 Å-thick slice from the RMC-refined model, shown in (**h**). **i** Superposition of TEM simulation and atomistic model, highlighting the ordered regions in the simulation. The particle clarity in (**g**) is highly sensitive to particle orientation, hence some ordered regions, highlighted in (**i**), are clearer than others.

**Fig. 3:**
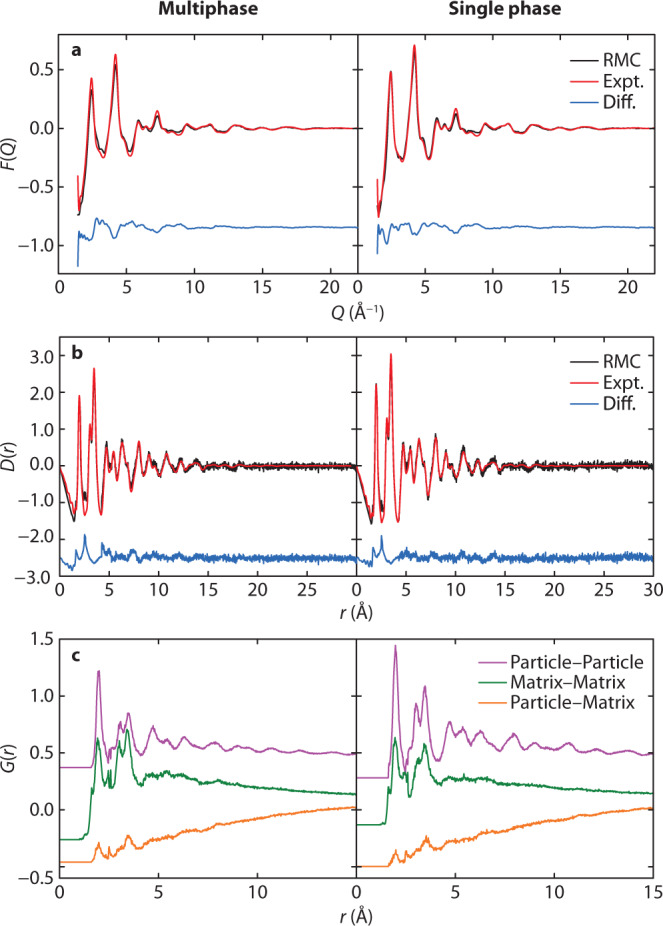
RMC fits to ferrihydrite models. **a** RMC fits to reciprocal space data *F*(*Q*) and **b** the PDF, using the *D*(*r*) normalisation—see ref. ^70^ for details. **c** Individual contributions to the PDF from particle–particle, matrix–matrix, and particle–matrix correlations, shown in the *G*(*r*) formalism; *G*(*r*) = *D*(*r*)∕4*πrρ*_0_, where *ρ*_0_ is the sample number density. Particle–particle and matrix–matrix correlations are offset by 1.5 and 0.15 units in *G*(*r*), respectively, for clarity.

**Fig. 4:**
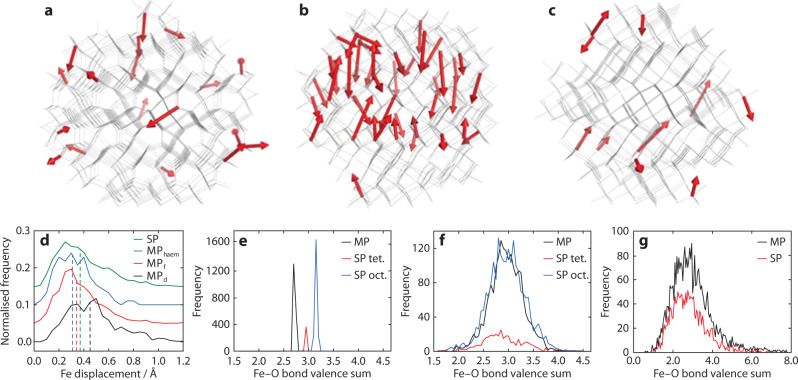
Fe displacements and bond valence sums. Representative Fe displacement vectors for **a** the single-phase model, **b** defective and **c** non-defective particles in the multiphase model. Only the largest displacements (>0.8 Å) are shown; vector magnitudes are scaled up to emphasise visually the direction of cation movement. The grey wireframes show the initial atomic coordinates. **d** Histogram of Fe displacements across all particles in the simulation box—frequencies are normalised against the number of cations in each type of particle. ‘Single phase’ and ‘multiphase’ are abbreviated to ‘SP’ and ‘MP’ in the plot legends. **e** Calculated Fe–O bond valence sums for the nanoparticles prior to RMC refinement, **f** post-refinement, and **g** for 4,5, and 6 co-ordinate matrix Fe cations (73% of Fe in multiphase, 64% in single phase), post-refinement. The contributions from tetrahedral and octahedral Fe in the single-phase model are calculated separately in (**f**). The standard formula $V=\sum \{\exp [({R}_{0}-{R}_{i})/b]\}$ is used with the literature values *b*  = 0.37, *R*_0_ = 1.76 Å, and a maximum Fe–O bond length of 2.6 Å was allowed for the value of *R*_i_.

We carried out RMC refinements using these nanocomposite models as our starting configurations. During these refinements, atom positions were adjusted in order to maximise the quality of fit to both X-ray PDF and reciprocal space *F*(*Q*) data. A representative atomistic model obtained from these refinements is shown in Fig. 2 and the corresponding fits to data are shown in Fig. 3; full details are given in Supplementary Note 2 and Supplementary Figs. 2–5. What is immediately obvious is that the quality of RMC fit, in both real (PDF) and reciprocal-space (*F*(*Q*)) is comparable for both models; certainly the differences in fit are smaller than our uncertainty in the experimental data. Taken at face value, both models appear consistent with the X-ray scattering data; however, by examining the particle restructuring required in order to achieve this fit, we now show that only the SP model accurately describes the structure of ferrihydrite. In Fig. 4a–c, we illustrate the most severe structural reorganisations that occur in representative ferrihydrite nanoparticles of the three different types contained within our RMC models: namely, the single nanoparticle component of the SP model and the d- and f-phases of the MP model. Whereas for the f-phase and the SP model the largest structural reorganisations occur at the nanoparticle/matrix interface (consistent with experiment^43,44^), our models of the d-phase nanoparticles systematically exhibit large internal reorganisation processes with collective Fe displacements >0.8 Å.

We quantify this observation in Fig. 4d, where the distribution of Fe reorganisation magnitudes is given for all four nanoparticle types across our ensemble of RMC configurations. The d-phase nanoparticles of the MP model are conspicuous in having a greater proportion of large Fe displacements. The driving force for this reorganisation is partly an incompatibility between the underlying nanoparticle structure and the data, and partly also the presence of short Fe…Fe contacts in the original model. When constructing our RMC starting configurations, it became clear that the rules describing cation distributions in the MP model of ref. ^23^ are in some respects internally inconsistent; these inconsistencies are obfuscated by a description in terms of conventional unit cells (further discussion is given in Supplementary Note 1). The most significant occurs in the d-phase, where a disordered stacking sequence gives unphysically short Fe…Fe contacts (1.8 Å) whenever equivalent layers occur in succession. A schematic diagram illustrating this point is given in Supplementary Fig. 1.

So we find that the MP model cannot account at once for both PDF and *F*(*Q*) data without substantial structural reorganisation; that is to say the MP crystallographic unit-cell description of 2-line ferrihydrite cannot be correct. By contrast, the SP model requires only a relatively subtle distortion to show consistency with the data, which suggests the model provides the more realistic representation of the structure of 2-line ferrihydrite. We have reached this conclusion by building what is (to the best of our knowledge) the first atomistic configuration of the MP model, free of the simplifications that have to date prevented fair and direct interrogation by other approaches.

### Nanocomposite model physicality

We proceeded to check for any unphysical local structure in either RMC refinement, in order to assess the efficacy of our large-scale nanocomposite modelling approach. The partial PDFs that describe particle–particle, matrix–matrix, and particle–matrix correlations are illustrated in Fig. 3c; these show varying degrees of structured correlation as a function of distance, and all have a chemically-intuitive form. Particle–particle interactions are most strongly correlated for *r* ≲ 10 Å and continue to contribute the most structure to the overall PDF over longer distances up to the particle diameter. Structured correlations are only evident within the matrix below 5 Å, showing that local atomic environments are still well-defined in an otherwise apparently-amorphous material. At larger distances, the matrix contribution to the PDF is unstructured and so this component does not compensate for a poorly-fitting particle model by contributing to unreasonable regions of the PDF. We note that the distinction between particle and matrix correlation functions illustrates the need to include a matrix component explicitly when fitting the PDF rather than relying on empirical modifications to the particle–particle correlation functions. In many ways the matrix–particle correlations are qualitatively similar to the matrix–matrix correlations: structure at low-*r* reflects well-defined chemical order at the particle–matrix interface and the smoothly-continuous behaviour at high-*r* shows the absence of structured correlations at distances  ≳ 5 Å. So there is no evidence that our refined RMC configurations contain any unphysical correlations; we provide a more detailed interpretation of the pairwise correlation functions to support this statement in Supplementary Note 2 and Supplementary Figs. 4–5.

### Addressing historical objections to the single-phase model

That RMC refinements based on both SP and MP models give comparable fits to data and correspond to similar particle–particle, matrix–matrix, and particle–matrix correlations answers a key point of contention in the literature. In earlier studies where damped periodic models had been used to interpret X-ray scattering patterns, it was thought that neither the SP nor the MP model could account simultaneously for both real-space and reciprocal-space transforms of the data^26^. Here we have shown that, by *e.g*. allowing relaxation at the nanoparticle/matrix interface, the SP model is consistent with the interatomic separations measured in the PDF and also the signature of periodic structural features reflected directly in the diffraction pattern. The same is true of the RMC-refined structure using the MP model particles, although distortions greater than ‘relaxation’ are required, to the extent that the final structure is not representative of the original MP model description.

A further historical objection to the SP model concerns its ability to reproduce chemically sensible Fe bond valence sum (bvs) values^24,45^. We are able to extract bvs distributions from our RMC configurations and hence address this point directly. In our analysis we define the bvs as $V=\sum \{\exp [({R}_{0}-{R}_{i})/b]\}$ where standard values *b* = 0.37, *R*_0_ = 1.76 Å are used^46^. For a given Fe atom, any Fe–O contact < 2.6 Å was used as an *R*_i_ value. Figure 4e shows the distribution of Fe bvs values for the three ferrihydrite nanoparticle starting configurations; note that these are essentially delta functions because the nanoparticles are generated based on fixed atom coordinates taken from periodic models. Following refinement, the bvs distributions broaden (Fig. 4f); the widths we observe are entirely consistent with other RMC studies^47^ and are to be expected for real systems where there are both thermal and configurational fluctuations. It is the midpoint of these distributions that is physically meaningful, and we find that both MP and SP models arrive at bvs values centred around 3.0 Å, as expected. That the Fe bvs values converge on a similar distribution in both MP and SP models—despite the differences in initial bvs distributions—is a strong indication that the data themselves are sensitive to a single robust set of bond valence parameters. Moreover, the bvs distribution for the matrix component also peaks at a physically sensible value  ≃3.0, albeit with a greater variance (Fig. 4g).

### Restructuring in the single-phase model

**Fig. 5:**
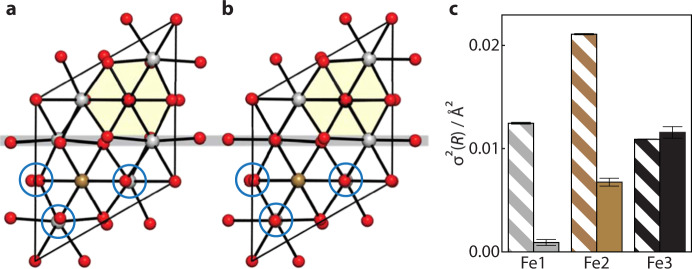
Revised unit-cell model for 2-line ferrihydrite. Projection along the *c*-axis showing the average unit cell coordinates for **a** the SP starting model (ref. ^24^) and **b** our newly-refined RMC refinements. Atom colours follow the scheme of Fig. 1d. Regions of each cell are highlighted to emphasise the decrease in structural distortion that follows RMC refinement. **c** Quantification of Fe coordination polyhedral distortions, as determined from the tensorial principal axis variances in pieface^48^, for starting (striped bars) and RMC-refined (filled bars) models. Fe1 and Fe2 are octahedral sites; Fe3 is the tetrahedral site. Error bars indicate the standard uncertainty on the mean variance.

Having established that our RMC-refined SP model provides the more physical description of the experimental PDF of ferrihydrite, we sought to extract from our RMC configurations the most succinct structural description of the nanocrystalline component against which we might compare the original akdalaite-based models of refs. ^10,24^. To this end, we first projected all atomic positions within a single nanoparticle onto a single unit cell, and repeated this calculation for each particle in our RMC box (accounting for differences in particle orientation, of course). For a given nanoparticle we found an entirely reasonable distribution of atomic positions, with no sign of anomalously large displacements. The average atomic positions obtained showed excellent consistency from particle to particle (see Supplementary Fig. 6). And so we could extract a set of averaged RMC-refined atomic coordinates for comparison against the small-box-model values given in ref. ^24^; these coordinates are compared in Table 1. Further details regarding atomic displacement distributions and consistency checks are given in Supplementary Note 3, Supplementary Table 5, and Supplementary Figs. 6, 7. While the difference in structures is relatively subtle, we found the RMC model to give less distorted Fe coordination geometries (Fig. 5). We quantified this observation using the minimum bounding ellipsoid method, as implemented in the program Pieface^48^. Here the degree of distortion is related to the asphericity of the smallest ellipsoid that can encompass a given coordination polyhedron. The effect of the RMC fitting is to drive both crystallographically-distinct, six-coordinated Fe sites to adopt more ideal octahedral geometries (a more spherical ellipsoid), whereas the degree of distortion at the tetrahedral Fe site is essentially unchanged from that of the small-box model.

**Table 1:** Atomic coordinates for the SP model of 2-line ferrihydrite.

Atom	Wyckoff	occ.	RMC			Small-box		
	Position		*x*	*y*	*z*	*x*	*y*	*z*
Fe1	6*c*	1.0	0.1666	0.8334	0.3789	0.1642	0.8358	0.3739
Fe2	2*b*	0.5	$\frac{1}{3}$	$\frac{2}{3}$	0.6733	$\frac{1}{3}$	$\frac{2}{3}$	0.6726
Fe3	2*b*	0.5	$\frac{1}{3}$	$\frac{2}{3}$	0.0615	$\frac{1}{3}$	$\frac{2}{3}$	0.0617
O1	2*a*	1.0	0	0	0.0106	0	0	0.0255
O2	2*b*	1.0	$\frac{1}{3}$	$\frac{2}{3}$	0.2604	$\frac{1}{3}$	$\frac{2}{3}$	0.2579
O3	6*c*	1.0	0.1673	0.8327	0.7669	0.1772	0.8228	0.7802
O4	6*c*	1.0	0.5081	0.4919	0.0115	0.5136	0.4864	−0.0008

Atomic coordinates as determined using RMC (this study) and small-box modelling (ref. ^24^) of X-ray PDF data. The corresponding hexagonal unit cell has *P*6_3_*mc* space group symmetry and lattice parameters *a*  =  5.902 Å, *c*  =  9.255 Å.

## Discussion

The RMC-SP model accounts for the X-ray PDF and *F*(*Q*) data, but is it consistent with other measurements of 2-line ferrihydrite? In Fig. 2d, g–i, we compare qualitatively against electron microscopy images not used in the refinement of our RMC model. We find good agreement between images simulated from a representative 10 Å-thick slice of the RMC configuration (Fig. 2g–i) and experiment (Fig. 2d and Supplementary Fig. 8)—lattice fringes and particle outlines are distinguishable from surrounding amorphous content; regions in which there is no clear signature of ordering. Hence our RMC model provides a sensible representation of the structure of 2-line ferrihydrite at both atomic and nanometre scales.

Although constructed from a single nanocrystalline phase, the SP model we develop nevertheless contains three physical components: the ordered nanoparticle interiors, the disordered matrix, and the nanoparticle/matrix interface. The complexity even of this ‘simple’ nanocomposite helps explain some ostensibly contradictory experimental observations. For example, the partitioning of water, hydroxide, and even tetrahedral or octahedral iron is likely to vary amongst these three components, and different experimental probes will show different sensitivities to each component. The intensity of Bragg-like features in X-ray (and neutron) scattering patterns will be sensitive to the composition of the nanoparticle interior and not to that of the matrix. The importance of structural relaxation at nanoparticle boundaries is now increasingly recognised within the field^44,49^. The interest is motivated by the surface role in ferrihydrite’s geological significance—it sequesters other elements in the natural environment^2,50^ as well as forming the interface between water pockets and Fe-containing material^44,51–54^. Naturally, the X-ray PDF data we have used in our own study are simply not sufficiently sensitive to the location of hydrogen atoms, especially in the presence of strong scatterers such as Fe (as in all other X-ray studies of ferrihydrite^24^). Nevertheless, our model provides a starting point for subsequent computational determination of the likely partitioning of oxide, hydroxide, and water throughout each of the three nanocomposite components. An obvious target for future studies is the subsequent energetic relaxation of such models, but doing so involves addressing the enormous challenge of extending ab initio methods to large supercell configurations.

Given the paucity of information contained within the scattering functions of disordered and nanocomposite materials, and the enormous number of degrees of freedom available within an RMC refinement, it is unrealistic to expect RMC to arrive at a unique structural ‘solution’ during refinement^55,56^. The case of ferrihydrite is a fundamentally trickier problem than that of local structure determination in complex crystalline materials, where the presence of an underlying crystal lattice ensures richer information content in scattering data and imposes strong constraints on refinement^17^. Moreover, nanocomposite materials are inherently non-equilibrium phases, which in turn complicates the application of conventional computational methodologies to structural modelling. What we demonstrate here is that atomistic refinement approaches such as RMC can be used to test proposed models even for structurally complex nanocomposite phases. In the specific case of ferrihydrite we are able to identify an improved unit-cell description of its key nanocrystalline component and characterise the coherence lengthscale for structural correlations at the nanocrystal–matrix interface.

Crucially, the ability to model heterogeneous nanocomposites has significance well beyond the example of ferrihydrite, as important as this system may be. For example, there are closely-related geologically- and biologically-relevant materials for which the approach we develop here is likely to offer significant insight: the structure and transformations of amorphous calcium phosphate, a key precursor in bone formation^57–60^; crystallite nucleation in supramolecular gels^61^; nanodomain evolution in aqueous aerosols^62^; and the stabilising effects of porous functionalised silica on surrounding media^63^. Likewise, key components used in functional devices, such as battery cathodes, routinely contain mixtures of amorphous and nanostructured components and are not well described in terms of single-phase approximants^64^. We anticipate that nanocomposite structure refinement will be particularly important for the many cases where the interface between nanoparticle components—e.g. the amorphous matrix for ferrihydrite—is crucial for material function, since this is precisely the component unaccounted for by conventional multi-phase modelling^65^.

## Methods

### Synthesis of 2-line ferrihydrite

Two-line ferrihydrite was synthesised according to the method developed by Schwertmann and Cornell^66^. We note that the scattering and PDF evaluated as part of this study were published previously in Ref. ^67^ which includes additional details regarding sample synthesis.

### Total scattering data collection and processing

Total scattering X-ray data were collected on powdered dry ferrihydrite at beamline 11-ID-B (90.8 keV, *λ* = 0.13702 Å) at the Advanced Photon Source, Argonne National Laboratory. Additional details regarding the experimental setup can be found in ref. ^67^. The data were processed using GUDRUNX,^68,69^ correcting for background scattering, Compton scattering, multiple scattering and beam attenuation by the sample container, yielding the normalised total scattering function *F*(*Q*) and its Fourier transform—the PDF—using the *D*(*r*) normalisation with ${Q}_{\max }=22$ Å^−1^. The formalisms used here for the total scattering functions follow those outlined by Keen^70^. Owing to the lack of consensus on the accepted ferrihydrite model, the data were normalised differently for the MP and SP models, using the number density and element concentrations that were present in our atomistic models, omitting contributions from hydrogen. However, the different normalisations used resulted in only negligible differences to the data.

### Reverse Monte Carlo modelling

Atomistic models for RMC refinement were constructed using bespoke program code—full details are given in the Supplementary Methods. The cubic simulation box, with *a* = 60 Å, consisted of either nine SP particles or six MP-f, three MP-d and three haematite particles. RMCProfile was used to fit the configuration to the PDF and *F*(*Q*) data, simultaneously^31^. Owing to initial difficulties in fitting the PDF satisfactorily, the low-*r* region of the PDF was duplicated as an additional, third, dataset, effectively forcing the configurations to fit the shorter-distance local geometry more closely than required for large *r*. Closest approach constraints of 2.50, 1.64, and 2.50 Å were used for O–O, M–O, and M–M atom pairs, respectively. Pairs of M–O atoms that fell within the range 1.50–2.60 Å in the starting model were constrained to remain within this distance window. Refinement proceeded via random movement of randomly selected atoms, subject to distance-based restraints (see Supplementary Table 4). Moves were accepted if the fit to data improved or accepted within the probability $P=\exp (-\Delta {\chi }^{2}/2)$ if it worsened. Refinement proceeded for five days, after which no substantial improvement in data fit was observed.

### High-resolution transmission electron microscopy and image simulation

TEM specimens were prepared by pipetting small droplets of a fresh ferrihydrite suspension on lacey carbon film-coated 300 mesh Cu grids. The TEM images were obtained using a JEOL JEM 2100, operated at 200 kV. HRTEM images were simulated based on the multislice method using the MacTempas (Total Resolution) software. The diameter of the model nanoparticles was smaller than 60 Å and so the (60 Å)^3^ box of the RMC-refined model (Fig. 2a) was sliced in 10 Å increments for the multislice calculations. The calculations were performed at an acceleration voltage of 200 kV, spherical abberation of 1.2 mm, and defocus spread of 80 mrad. The thickness was set to 10 Å, i.e. the thickness of a single model slice. Simulated images with defocus values between  −600 and  +600 Å were compared with the experimental TEM images.

## Supplementary information

Supplementary Information

## Data Availability

The raw data on which this manuscript is based are openly available for download from 10.5287/bodleian:M8GnGvemj. These include the RMC configurations, real- and reciprocal-space fits, and the unit-cell model shown in Fig. 5.

## Code availability

The reverse Monte Carlo simulations were performed using RMCProfile, which is freely-available from http://www.rmcprofile.org/. Specific restraints used during the RMC process are given in Supplementary Table 4. All custom code used in this study was developed using widely available algorithms. Copies of the code used can be obtained upon request.
