# Supplementary material for: Nanocomposite structure of two-line ferrihydrite powder from total scattering
Source: Commun Chem. 2020 Feb 21;3:22. doi: 10.1038/s42004-020-0269-2 (PMC9814407; doi:10.1038/s42004-020-0269-2)
Supplement: Supplementary file 1 — Supplementary Information [file 42004_2020_269_MOESM1_ESM.pdf]

Nanocomposite structure of two-line ferrihydrite powder from total scattering

SUPPLEMENTARY INFORMATION

Nicholas P. Funnell,[1,2,*] Maxwell F. Fulford,[1,3] Sayako Inoué,[4]
Karel Kletetschka,[4] F. Marc Michel[4,*] and Andrew L. Goodwin[1,*]

[1] Department of Chemistry, University of Oxford, Inorganic Chemistry Laboratory,
South Parks Road, Oxford, OX1 3QR, U.K.

[2] ISIS Neutron and Muon Facility, Rutherford Appleton Laboratory,
Didcot, OX11 0QX, U.K.

[3] Department of Physics, Kings College London, Strand
London, WC2R 2LS, U.K.

[4] Department of Geosciences, Virginia Tech, Blacksburg, VA 24061, U.S.A

*To whom correspondence should be addressed;
E-mail: nick.funnell@stfc.ac.uk, mfrede2@vt.edu, andrew.goodwin@chem.ox.ac.uk.

Supplementary note 1; the multiphase model

The model described by Drits *et al* is a three-component mixture, consisting of a defect-free 'f' phase, a defective 'd' phase and small, cylindrical (ca. 10 Å diameter $\times$ 14 Å length) particles of haematite.[1] The f:d ratio is 2:1, and these are found in the volume ratio 3:1 with haematite, leading to the overall ratio 6:3:3 for f:d:haematite.

The f-phase consists of hexagonal stacks ($a = 2.96$ Å$, c = 9.40$ Å$, P\bar{3}1c$) of edge- and corner-sharing, octahedral Fe that follow the non-defective repeating sequence AcBcAbCb, where upper case letters identify O or OH sites, and lower case, Fe. Atomic coordinates are given in Supplementary Table 1. Fe sites are occupied with 50% probability and are displaced along the *c*-axis towards B and C layers. The B and C sites are reported to be occupied by O atoms 85% of the time, and water molecules for the remaining 15%, which form channels within the particle. However, the presence of water nanodomains means there cannot be any coordinating cations to these sites. On building a model, it became clear that the Fe occupancy must fall below 50% to satisfy the constraints of the water concentration; its exact value is dependent on the size and spatial distribution of the water nanodomains. Rather than presume an Fe occupancy when introducing water molecules into our model (using a Monte Carlo procedure) we opted to fix the Fe occupancy at the 50% literature value—as observed by X-ray diffraction, which would show greater sensitivity to ordered Fe than the relatively disordered O/H—preventing the formation of water channels in B and C-layers. Random occupancy of the b1/b2 and c1/c2 cation sites can, however, lead to uncoordinated oxygen ('water') atoms in A-layers. In the interest of including a small quantity of water in the model, we allowed this to occur—partial O/OH occupancy at A-sites has also been suggested by others.[2]

The d-phase is comprised of a random AbCb and AcBc layer sequence, with atom coordinates (reproduced in Supplementary Table 2) best described by a $P1$ hexagonal supercell, with respect to the *ab*-plane, with dimensions $a = 5.126$ Å$, c = 4.70$ Å. The Fe site occupancies are specific for each independent Fe site but still total to 50% overall for the whole unit cell. Drits *et al* state that A-site O atoms at *z* = 0 are half-occupied, however it becomes clear on construction of a physical model that a combination of half-occupancies in both the Fe and A-site O atoms would require some Fe atoms to have a coordination number lower than six. As identical layer fragments can occur in succession (i.e. AcBcAcBc or AbCbAbCb), this leads to the possibility of face-sharing octahedra along the *c*-direction, although each octahedron shares only one face. However, the occupancies of Fe coordinates with *z* = 0.19 (occ. = 0.4) and 0.81 (occ. = 0.8) on b or c-sites sites mean at least 20% of these sites must contain Fe simultaneously. The 4.70 Å length of the unit cell *c*-axis leads to these Fe atoms being separated by an unphysically short 1.786 Å. This is illustrated schematically in Supplementary Figure 1. We permitted these to exist in our starting model, keeping the occupancies fixed to literature values, but the subsequent reverse Monte Carlo refinement moves the atoms apart.

Supplementary note 1; the single phase model

The single-phase model, described by Michel *et al* is isostructural with akdalaite, $Al_{10}O_{14}(OH)_2$, having $P6_3mc$ symmetry and unit cell dimensions *a* = 5.91 Å, *c* = 8.95 Å.[3,4] There are three crystallographically distinct Fe atoms, one of which is a fully-occupied octahedral site, and the others partially occupied octahedral and tetrahedral sites. The partial occupancies are thought to vary with particle size and degree of crystallinity—we fixed these both to the lowest allowed value of 0.5, corresponding to the most disordered form of ferrihydrite. Atomic coordinates are given in Supplementary Table 3.

Supplementary methods; model construction

Construction of all particle models was carried out using bespoke FORTRAN90 code. First, randomly-positioned hard spheres with diameters of 30 Å were placed at non-intersecting locations in a $60 \times 60 \times 60\,\text{Å}$ cubic simulation box, filling approximately 50% of available space. This density is closely approaching the random close-packed limit of 64% for spherical particles.[5] Particles of the SP or MP structures, with diameters of ca. 27 Å were generated sequentially, and substituted for the randomly-positioned spheres in the simulation box.

In terms of individual particle construction—for the SP model—we used the coordinates from the average structure to create a supercell with dimensions greater than the desired nanoparticle size, setting each Fe2 and Fe3 site to be present with a 50% probability. Any atoms beyond a 29 Å diameter were then removed. Lastly, under-coordinated Fe atoms, and then O atoms, were removed, resulting in a particle with the desired dimensions and an outer shell of hydroxyl groups. The particle was randomly orientated and introduced to the simulation box, replacing one of the hard spheres.

MP particles were assembled layer-by-layer until the target dimensions were reached. In f-phase particles, this allowed us to use a Monte Carlo approach within each layer, moving Fe and O-atom vacancies at random, and minimising the difference from target occupancies, in an attempt to satisfy the complex requirements of the model and introduce water nanodomains. In the d-phase particles, a similar method was used to try and limit face-sharing of octahedra to one just face, however, after the Monte Carlo procedure, a few violations of this structural requirement were present due to the self-inconsistency of the model (with respect to Fe occupancies), described in the previous section. Cylindrical haematite particles were generated by simply creating an orthogonalised supercell of the known crystal structure; particle dimensions used were ca. 14 Å length and 10 Å diameter.[6] All three particle types were reduced to the desired particle size, and given sensible surface structures, in an identical manner to the SP particles.

To create the nanomatrix, a second simulation box was filled with randomly-placed Fe and O atoms, using the dwbuild module in RMCProfile,[7] subject to the following minimum-approach distance constraints: Fe–Fe, 2.0 Å; Fe–O, 1.0 Å; O–O, 1.5 Å. The overall simulation box density was set to closely match experimental values. The disordered atoms were then moved via a Monte Carlo algorithm to more chemically-reasonable positions by using local distance restraints, minimising the difference between the average of each of the six shortest Fe–Fe, O–O, Fe–O, and O–Fe distances in the matrix (noting that Fe–O $\not\equiv$ O–Fe), i.e.

$$\chi^2 = \sum_n (\langle d\text{model}_n \rangle - d\text{target}_n) \qquad (1)$$

where n is in the range 1–6. The crystalline, average structure of the SP model was used to derive target distances for the SP matrix. The equivalent procedure was not possible for the MP model; attempts to do so resulted in large O-rich (water) regions. Given its close structural relationship, haematite was instead used to obtain target distances. Following refinement, the particle and matrix boxes were then superimposed, and any matrix atoms that overlapped or lay within an atom-pair-specific distance (Fe–O, 1.7 Å; O–O, 1.8 Å; Fe–Fe, 2.0 Å) of the particle were removed.

Supplementary methods; reverse Monte Carlo refinement

The models described above were used as the starting point for RMC refinement.[8] The RMC approach proceeds via random movement of individual atoms, minimising the fits to pair distribution function $D(r)$ and total scattering structure factor $F(Q)$ data, defined as:

$$\chi^2_{D(r)} = \sum_j [D_{\mathrm{calc}}(r_j) - D_{\mathrm{exp}}(r_j)]^2 \sigma^{-2}_{D(r)} \quad (2)$$

$$\chi^2_{QF(Q)} = \sum_j [QF_{\mathrm{calc}}(Q_j) - QF_{\mathrm{exp}}(Q_j)]^2 \sigma^{-2}_{QF(Q)} \quad (3)$$

where σ is a relative weighting parameter for each dataset. Minimum approach constraints were imposed on individual atom pairs—these values were set based on peak minima and maxima in the PDF data. Additional distance window (DW) constraints were used to define the range over which atom pairs could be separated from each other, the pairs being predefined by a 'connectivity' list, with limits set by the distance window range. Some DW values were set below those of the closet approach constraints, to ensure that bonded atoms in the starting models were included in a DW, otherwise these would be unconstrained during the course of refinement. The values used for all distance constraints are given in Supplementary Table 4. All RMC refinements were carried out with RMCProfile over 5 days, after which no further improvements in the fit to data were observed.[7] Partial pair distribution functions for each atom pair are shown in Supplementary Figures 2 and 3.

Supplementary Note 2

Partial pair distribution functions for each atom pair in the refined MP and SP models are shown in Supplementary Figures 2 and 3, respectively, with the $g(r)$ formalism. In both models, all particle–particle interactions (inter and intra) are consistent with the typical form of a nanoparticle PDF; they possess well-defined correlations at low *r* that diminish rapidly as distance increases, reflecting the finite size of the particles. In the MP model, there is a small Fe–Fe peak situated at ca. 2 Å—this is likely a remnant of the few unphysically-short Fe–Fe contacts present in the starting model (see discussion in Supplementary Note 1) that have been unable to move any further apart over the course of refinement. The relative proportion of Fe–Fe interactions that fall within this region though, is very small.

Structured correlations between atoms within the matrix are present only up to ca. 5–8 Å—in all cases there is an intense low-*r* peak that mirrors the position of the corresponding peak in the particle–particle $g(r)$'s, followed by less-pronounced features up to ca. 8 Å. There are no further structured correlations beyond this distance. Creating and refining a chemically-plausible composition and structure for the matrix was challenging: the semi-random initial placement of the atoms, their packing density, and the need to refine their positions against the PDF led to some atoms becoming 'stuck'. These are evident in the very narrow sharp spikes in the $g(r)$'s, found at the limit of the DW constraints—the most obvious being present between oxygen atoms, indicating a level of order that is unlikely to actually be present in the ferrihydrite sample. However, the similarity in peak position to those in the particle $g(r)$'s (Supplementary Figures 2 and 3), the overall agreement with the PDF $D(r)$ (Figure 3), a visual inspection of local geometry (Figure 2), and the distribution of Fe–O bond valence sums (Figure 4) all suggest that the majority of the refined structure is broadly sensible, and consistent with the data. We reiterate that we are not aiming to suggest what the 'true' structure of the non-crystalline component is.

Turning to the correlations between the particles and matrix—the most striking feature is the large underlying background contribution in the $g(r)$'s which can be ascribed to the unusual geometry of the RMC refinement box: a heterogeneous (nanocomposite) system with particle spheres embedded in another phase, that results in density fluctuations across the box. The particles are approximately 27 Å in size and so atoms toward the centre of the particle (i.e. 13.5 Å) do not start to contribute to the $g(r)$'s until this distance is reached—intensity increases with the number of pairwise correlations n_{ij}, according to the relationship:

$$g(r) = \frac{n_{ij}}{4\pi r^2 \mathrm{d}r \rho_j} \tag{4}$$

where ρ_j corresponds to the number density of particle j. Thus the turning point in diffuse intensity is strongly related to the size of the particles. Very sharp peaks in the $g(r)$'s are present for the same reasons as discussed above.

Lastly, we address the possibility that the matrix atoms of each model could act as a means of inappropriately accounting for structured correlations in the overall PDF. Direct comparison between the two models is complicated by the different nanoparticle compositions between the two models as well as the different nanoparticle:matrix ratios. This leads to a small difference in the normalisations that are used on the data. We reproduce Figure 3 from the main manuscript here (as Supplementary Figure 4) and normalise correlations involving matrix atoms by $-G(r=0)$, to place them on a comparable scale. Supplementary Figure 5 shows matrix–matrix, and particle–matrix, correlations in the two models. What is immediately clear is that the two sets of correlations are very similar. The most pronounced differences occur within the matrix components over the region 1.6–2.5 Å—covering the range of nearest-neighbour Fe–O bond lengths. However, as the local atomic environments of the SP and MP matrices are based on the respective structures of their ordered ferrihydrite component, we would reasonably expect there to be

differences in local coordination. More important are the remaining strong structured correlations, which persist until approximately 4.5 Å—these are highlighted in yellow in Supplementary Figure 5. It is evident that there are only very subtle differences between the structured correlations in the matrices of the two models, indicating that in neither model is the matrix over/undercompensating for excess/insufficient particle structuring.

Supplementary Note 3

Averaging over RMC-refined positions in the SP model revealed increased ordering relative to the atomic starting coordinates (Figure 5 in the main manuscript). This arises predominantly from reduced distortion in Fe–O octahedra; the tetrahedra remain unchanged, within error. To calculate the degree of polyhedral distortion, we used PIEFACE (Polyhedra-Inscribing Ellipsoids For Analysing Crystallographic Environments)[9] which describes distortions in terms of the smallest ellipsoid that bounds the polyhedron. This approach thus considers both the effects of bond length and angle distortion simultaneously. We produce the program output in Supplementary Table 5. S is a dimensionless quantity that describes the sphericity of the ellipsoid: $S < 0$ is an oblate distortion, and $S > 0$ is prolate; $S = 0$ corresponds to a perfect sphere. The ellipsoids are defined with three principal atomic radii, and their average $\langle R \rangle$ is given in the Table. The standard deviation $\sigma(R)$ is a measure of distortion—for a completely regular polyhedron $\sigma(R) = 0$.

We also reproduce an enlarged version of Figure 4 here (as Supplementary Figure 7), showing that the RMC-refined bond valence sums converge on sensible values.

Supplementary Table 1: Atomic coordinates for defect-free, multiphase ferrihydrite particles, in $P\bar{3}1c$, $a = 2.96\,\text{Å}$, $c = 9.40\,\text{Å}$. Reproduced from Table 1 in Ref. 1

atoms	x	y	z	occ
O	0.0	0.0	0.0	1.0
Fe	1/3	2/3	0.15	0.5
O	2/3	1/3	0.25	0.85

Supplementary Table 2: Atomic coordinates for defective, multiphase ferrihydrite particles, with hexagonal supercell dimensions $a = 5.126\,\text{Å}$, $c = 4.70\,\text{Å}$. Reproduced from Table 3 in Ref. 1

	Ac_1Bc_2A					Ab_1Cb_2A				
atoms	site	x	y	z	occ.	site	x	y	z	occ.
O,OH	A	0.0	0.0	0.0	0.5	A	0.0	0.0	0.0	0.5
	A	1/3	2/3	0.0	0.5	A	1/3	2/3	0.0	0.5
	A	2/3	1/3	0.0	0.5	A	2/3	1/3	0.0	0.5
Fe	c1	2/3	0.0	0.19	0.4	b1	1/3	0.0	0.19	0.4
	c1	0.0	2/3	0.31	0.55	b1	0.0	1/3	0.31	0.55
	c1	1/3	1/3	0.31	0.55	b1	2/3	2/3	0.31	0.55
O,OH	B	1/3	0.0	0.5	1.0	C	2/3	0.0	0.5	1.0
	B	0.0	1/3	0.5	1.0	C	0.0	2/3	0.5	1.0
	B	2/3	2/3	0.5	1.0	C	1/3	1/3	0.5	1.0
Fe	c2	2/3	0.0	0.81	0.8	b2	1/3	0.0	0.81	0.8
	c2	0.0	2/3	0.69	0.35	b2	0.0	1/3	0.69	0.35
	c2	1/3	1/3	0.69	0.35	b2	2/3	2/3	0.69	0.35
O,OH	A	0.0	0.0	1.0	0.5	A	0.0	0.0	1.0	0.5
	A	1/3	2/3	1.0	0.5	A	1/3	2/3	1.0	0.5
	A	2/3	1/3	1.0	0.5	A	2/3	1/3	1.0	0.5

Supplementary Table 3: Atomic coordinates for single-phase, ferrihydrite particles with space group symmetry $P6_3mc$, $a = 5.91\,\text{Å}$, $c = 8.95\,\text{Å}$. Reproduced from Ref. 4, supplementary information

atoms	x	y	z	occ.
Fe1	0.1642	0.3284	0.3739	1.0
Fe2	2/3	1/3	0.1726	0.5
Fe3	1/3	2/3	0.0617	0.5
O1	0.0	0.0	0.0255	1.0
O2	0.5136	0.0272	0.9992	1.0
O3	0.8228	0.6456	0.2802	1.0
O4	1/3	2/3	0.2579	1.0

Supplementary Table 4: Closest approach (CA) and distance window (DW) constraints, where applicable, used for select atom pairs.

Atom pair	CA / Å	$DW_{min.}$ / Å	$DW_{max.}$ / Å
$Fe_{particle}$–$Fe_{particle}$	2.50	-	-
$Fe_{particle}$–Fe_{matrix}	2.50	-	-
Fe_{matrix}–Fe_{matrix}	2.50	-	-
$O_{particle}$–$O_{particle}$	2.50	-	-
$O_{particle}$–O_{matrix}	2.50	-	-
O_{matrix}–O_{matrix}	2.50	-	-
$Fe_{particle}$–$O_{particle}$	1.64	1.50	2.60
$Fe_{particle}$–O_{matrix}	1.64	1.50	2.60
Fe_{matrix}–$O_{particle}$	1.64	1.50	2.60
Fe_{matrix}–O_{matrix}	1.64	1.50	2.60

Supplementary Table 5: PIEFACE output for averaged Fe–O polyhedra in the SP model, using $P1$ symmetry. Fe1 sites are fully-occupied octahedra, Fe2 and Fe3 sites are partially-occupied octahedra and tetrahedra, respectively. The average for each Fe site is shown at the end of the relevant site block in bold.

	SP starting model				RMC-refined			
Site	S	$\langle R \rangle/\text{Å}$	$\sigma R/\text{Å}$	Vol. / $Å^3$	S	$\langle R \rangle/\text{Å}$	$\sigma R/\text{Å}$	Vol. / $Å^3$
Fe1	−0.127	2.003	0.144	33.374	−0.050	2.019	0.087	34.381
Fe1	−0.127	2.003	0.146	33.362	−0.034	2.023	0.079	34.617
Fe1	−0.127	2.003	0.145	33.364	−0.012	2.025	0.084	34.713
Fe1	−0.127	2.003	0.145	33.375	−0.033	2.016	0.072	34.261
Fe1	−0.127	2.003	0.145	33.382	−0.039	2.018	0.086	34.312
Fe1	−0.126	2.003	0.146	33.363	−0.044	2.019	0.085	34.379
**Average**	**−0.127**	**2.003**	**0.145**	**33.370**	**−0.035**	**2.020**	**0.082**	**34.444**
Fe2	−0.108	2.017	0.111	34.215	−0.013	2.016	0.025	34.309
Fe2	−0.109	2.018	0.112	34.252	−0.025	2.020	0.035	34.490
**Average**	**−0.108**	**2.017**	**0.112**	**34.233**	**−0.019**	**2.018**	**0.030**	**34.400**
Fe3	−0.113	1.883	0.105	27.846	−0.107	1.825	0.110	25.306
Fe3	−0.113	1.883	0.104	27.857	−0.104	1.819	0.105	25.065
**Average**	**−0.113**	**1.883**	**0.104**	**27.852**	**−0.106**	**1.822**	**0.108**	**25.186**

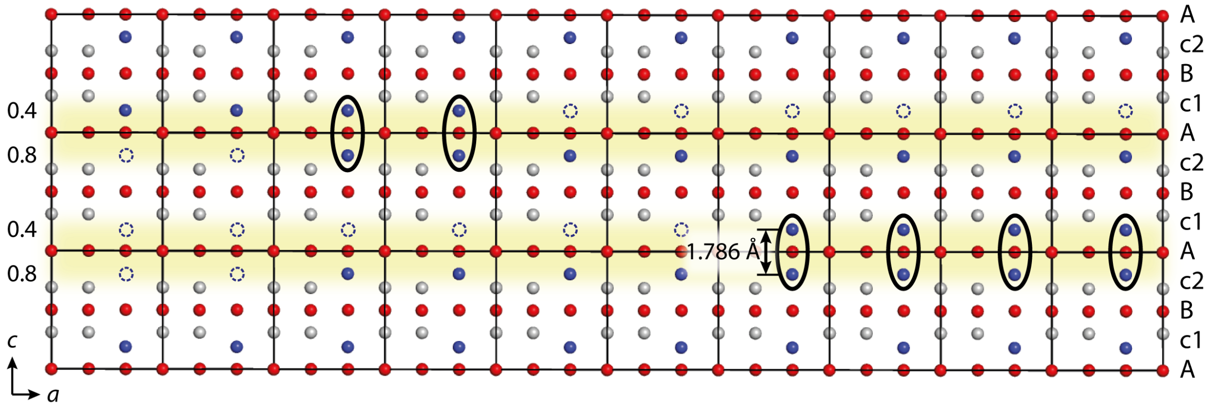

Supplementary Figure 1. d-phase schematic. Successive AcBc layers along the *c*-axis, as permitted to exist by the disordered layer-stacking sequence, shown over a $10a \times 1b \times 3c$ cell range. O atoms are shown in red, Fe in white, and 'close contact' Fe sites in blue. The respective occupancies of the pertinent Fe atoms are shown on the left-hand side, and these are reflected visually by filled and vacant sites—the latter indicated by open, broken circles. The choice of 10 cells along *a* enables straightforward reproduction of these occupancies. Two scenarios are shown by the shaded yellow areas; the top band is where the minumum possible number of short Fe$\cdots$Fe contacts occur (two), and the lower band the maximum (four). All short contacts are encircled. All other atoms are shown as being fully-occupied, although there are a number of other partial occupancies elsewhere in the cell.

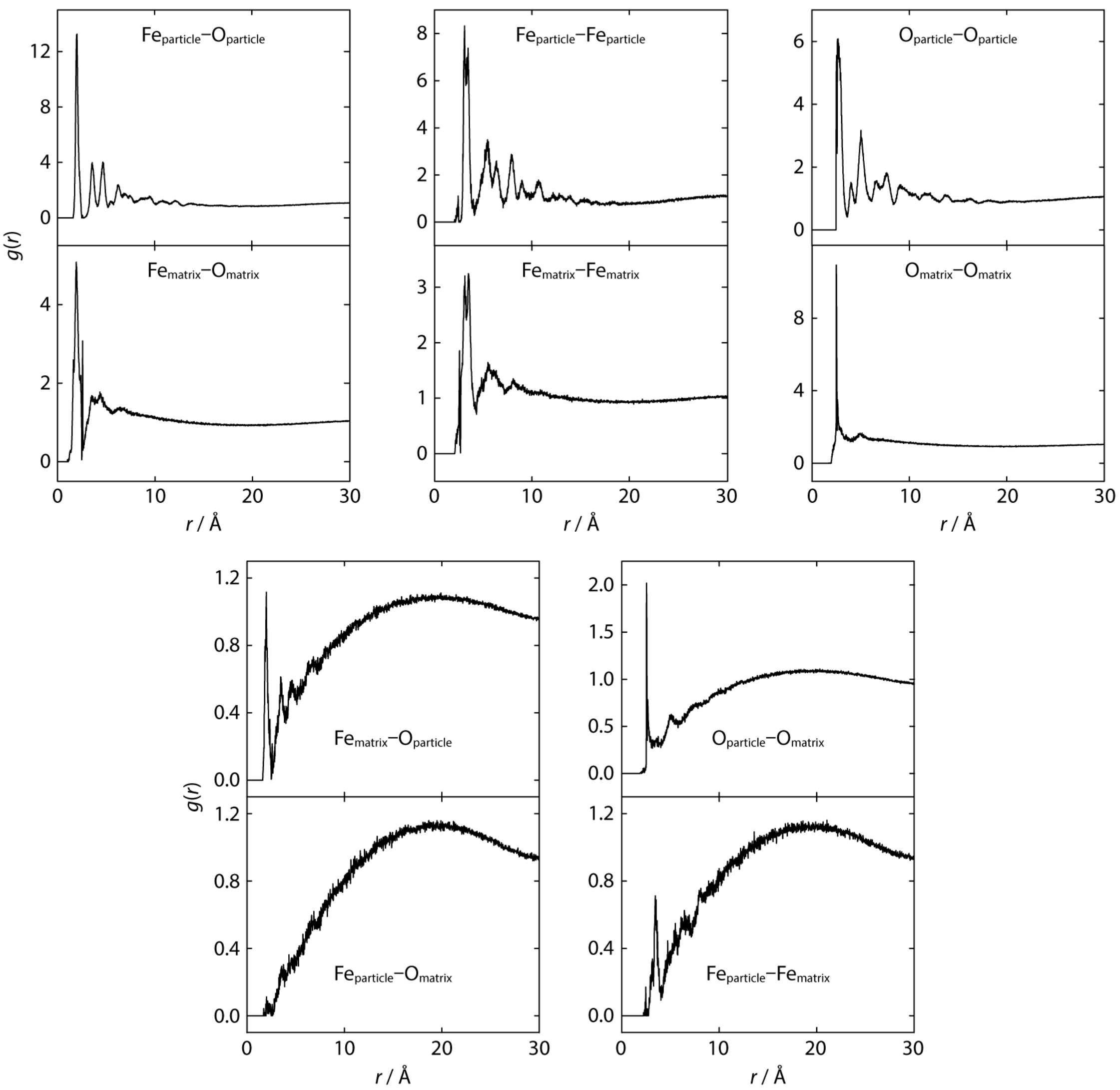

Supplementary Figure 2. MP model partial PDFs. Upper panels show partial pair distribution functions $g(r)$ for intra- and inter-particle atom-atom correlations, and intra-matrix correlations in the MP model. Lower panels show $g(r)$'s between matrix and particle atoms. Note that y-axes are not shown on the same scale.

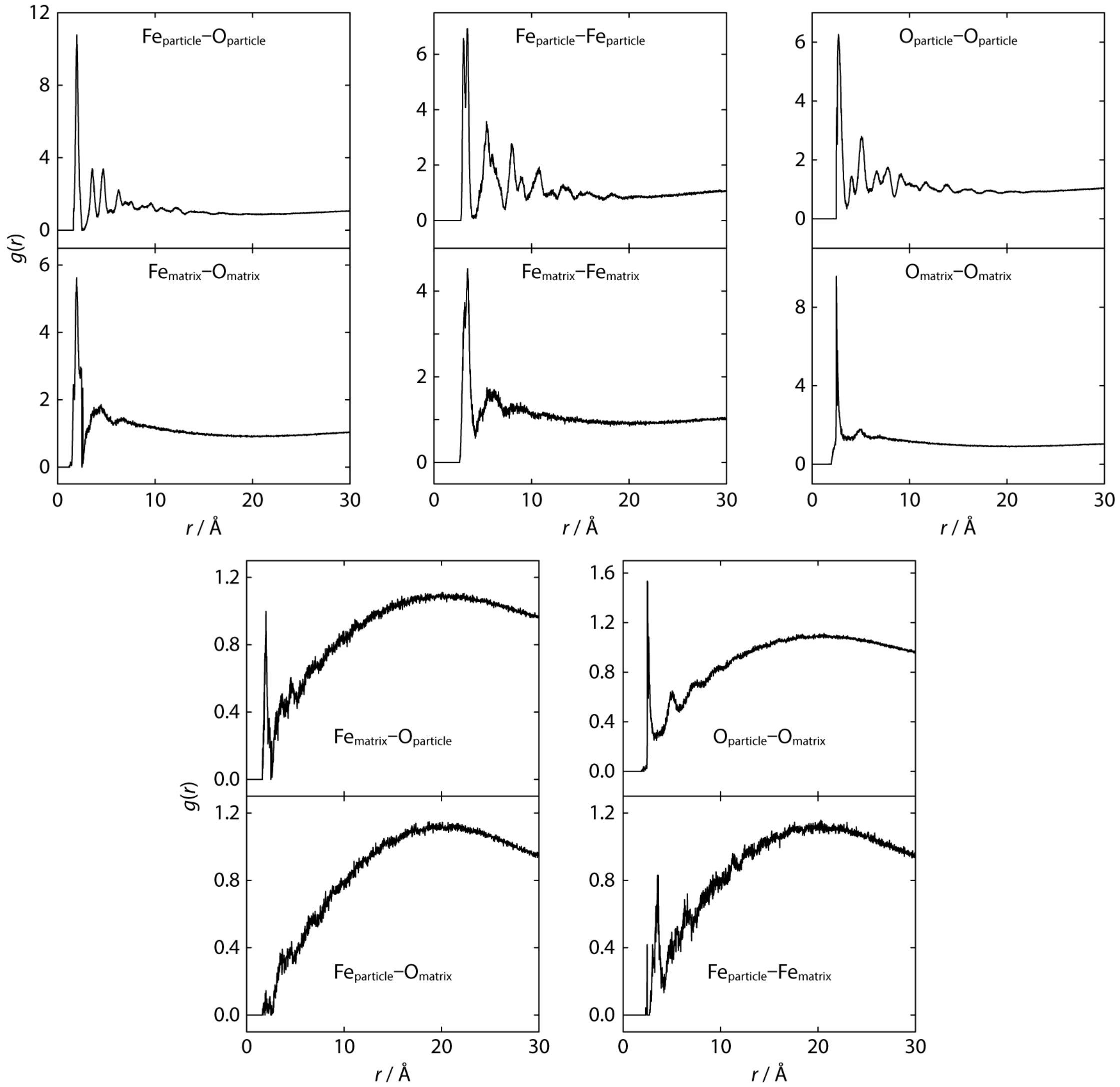

Supplementary Figure 3. SP model partial PDFs. Upper panels show partial pair distribution functions $g(r)$ for intra- and inter-particle atom-atom correlations, and intra-matrix correlations in the SP model. Lower panels show $g(r)$'s between matrix and particle atoms. Note that y-axes are not shown on the same scale.

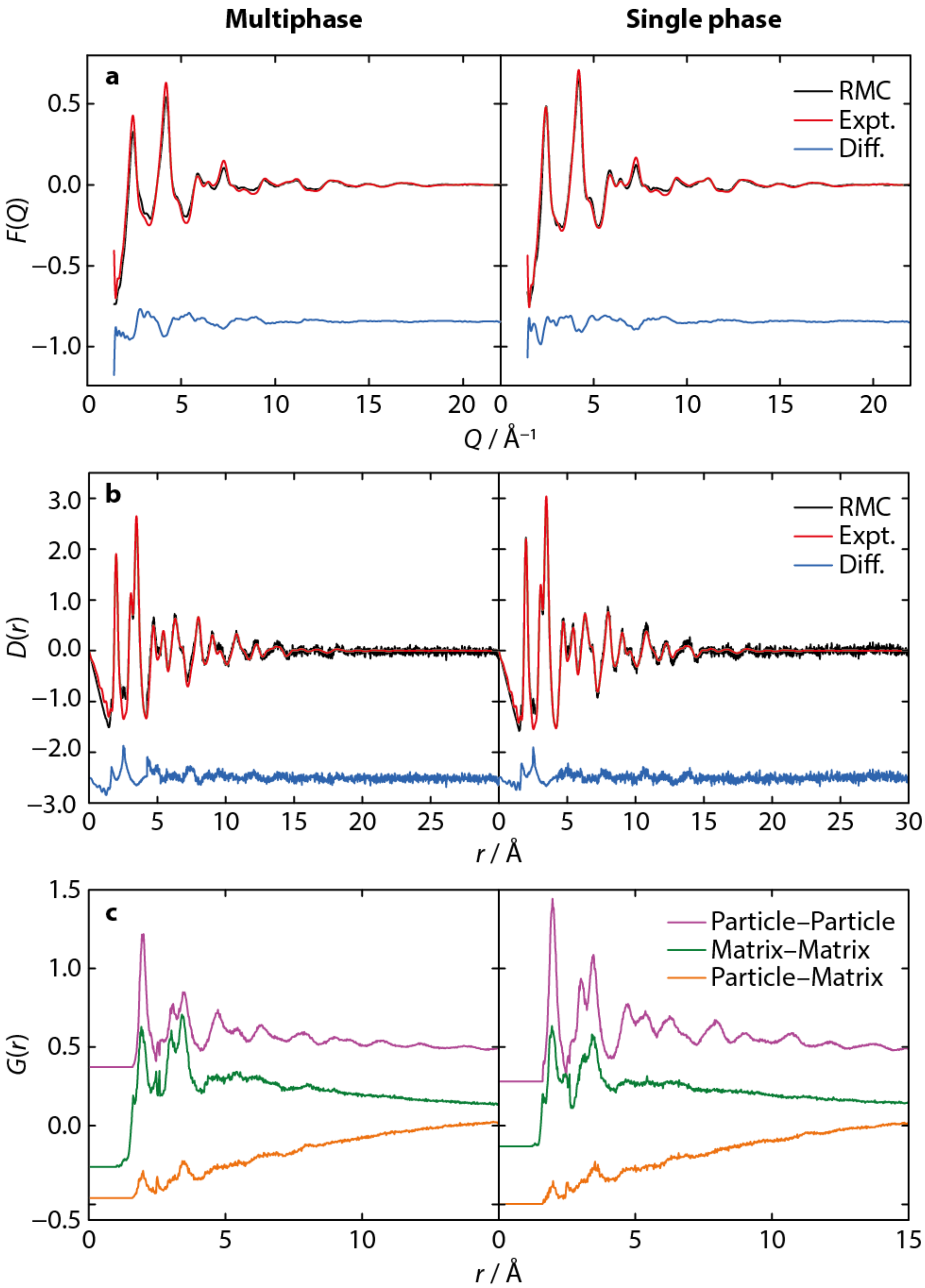

Supplementary Figure 4. RMC fits to ferrihydrite models. Reproduced from Figure 3 in the main manuscript. **(a)** RMC fits to reciprocal space data $F(Q)$ and **(b)** the PDF, using the $D(r)$ normalisation. **(c)** Individual contributions to the PDF from particle–particle, matrix–matrix, and particle–matrix correlations, shown in the $G(r)$ formalism. Particle–particle and matrix–matrix correlations are offset in y for visual clarity.

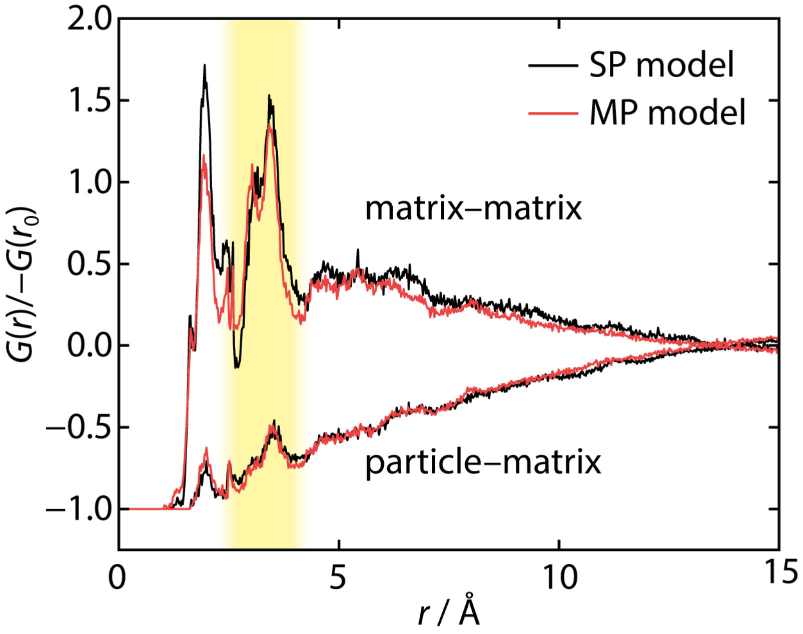

Supplementary Figure 5. Renormalised PDFs. PDFs for matrix–matrix (top) and particle–matrix (bottom) correlations for the SP and MP models. Yellow shading highlights the 2.5–4.0 Å region—distances which cover the structured correlations beyond immediate bonding environments.

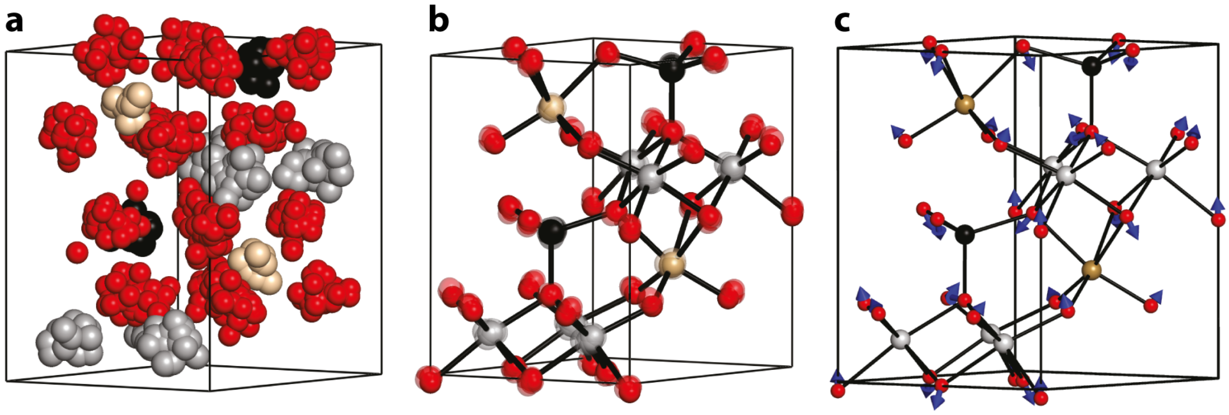

Supplementary Figure 6. RMC-refined SP model. (a) RMC-refined atomic distribution of a single SP particle, collapsed back onto a single unit cell, and **(b)** average refined positions for every SP particle (translucent atoms) all overlaid with atomic coordinates of the starting positions (solid atoms). Octahedral Fe1 and Fe2 atoms are shown in grey and brown, respectively; tetrahedral Fe3 atoms are shown in black. Overall oxygen atom trajectories **(c)**, averaged over all particles; displacement vectors are shown in blue with arrow lengths scaled by a factor of 2.5 for visual clarity.

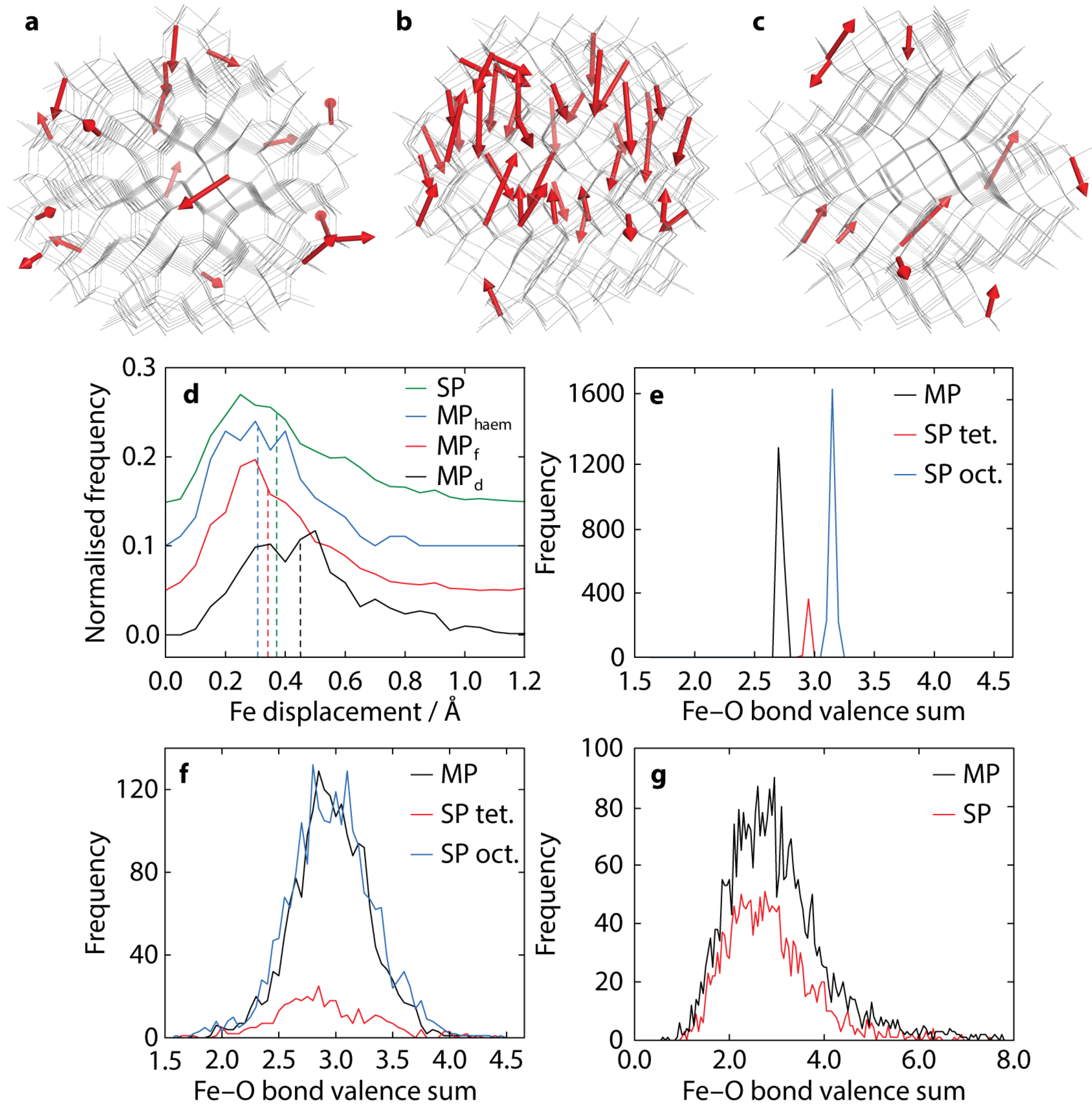

Supplementary Figure 7. Fe displacements and bond valence sums. Reproduction of Figure 4 from the main manuscript. Scaled Fe displacements (>0.8 Å) shown for **(a)** the SP model, **(b)** defective, and **(c)** non-defective particles in the MP model. **(d)** Histogram of Fe displacements across all particles, **(e)** calculated Fe–O bond valence sums for the nanoparticles prior to RMC refinement, **(f)** post-refinement, and **(g)** for 4, 5, and 6 co-ordinate matrix Fe cations (73% of Fe in the MP model, 64% in the SP) post-refinement.

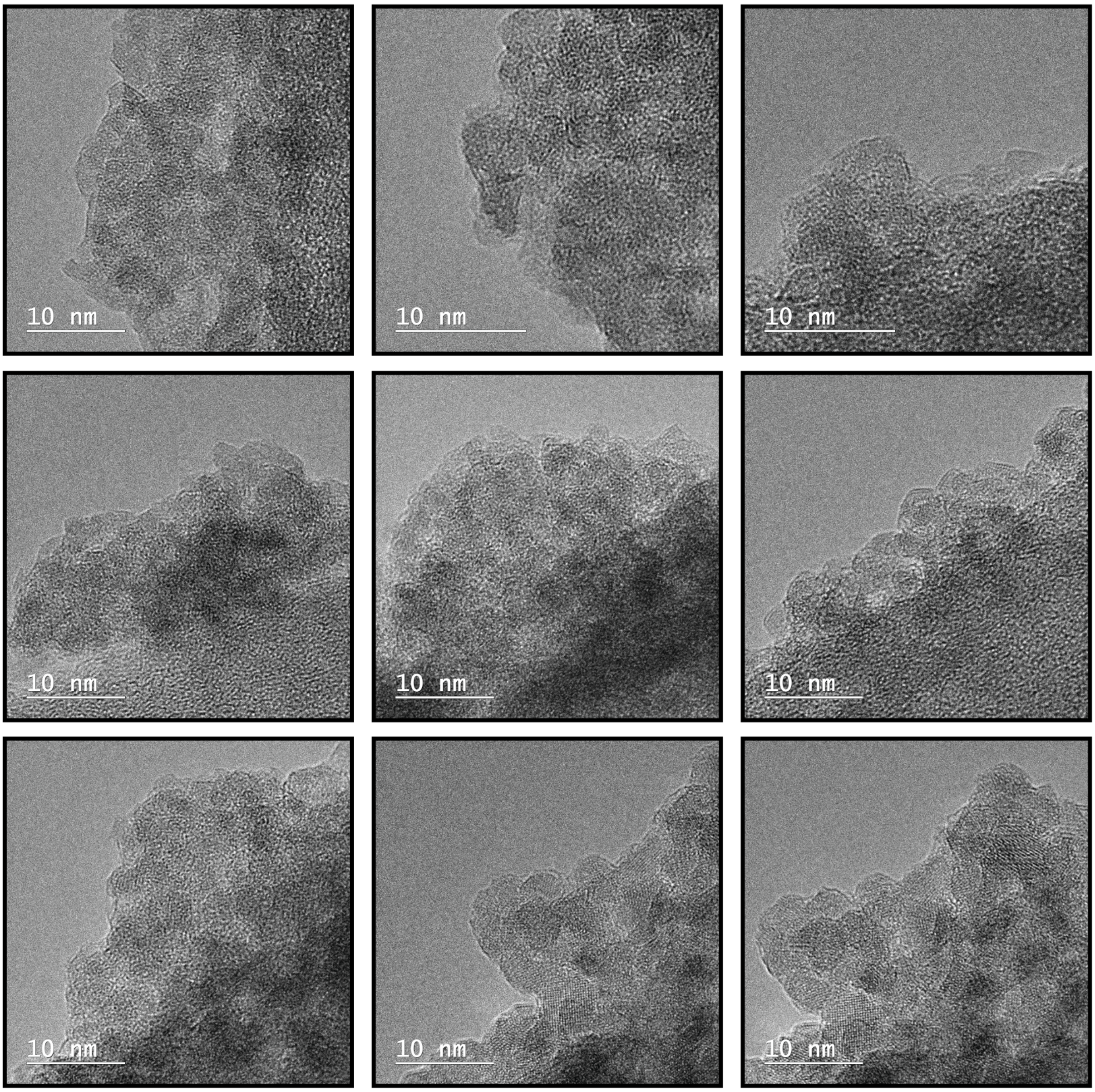

Supplementary Figure 8. TEM images. Representative transmission electron microscopy images of 2-line ferrihydrite. The scale bars differ slightly between images.

Supplementary References

(1) Drits, V. A., Sakharov, B. A., Salyn, A. L. & Manceau, A. Structural model for ferrihydrite. *Clay Miner.* **28**, 185–207 (1993).

(2) Jansen, E., Kyek, A., Schäfer, W. & Schwertmann, U. The structure of six-line ferrihydrite. *Appl. Phys. A* **74**, S1004–S1006 (2002).

(3) Michel, F. M. *et al.* The structure of ferrihydrite, a nanocrystalline material. *Science* **316**, 1726–1729 (2007).

(4) Michel, F. M. *et al.* Ordered ferrimagnetic form of ferrihydrite reveals links among structure, composition, and magnetism. *Proc. Nat. Acad. Sci.* **107**, 2787–2792 (2010).

(5) Torquato, S., Truskett, T. M. & Debenedetti, P. G. Is random close packing of spheres well defined? *Phys. Rev. Lett.* **84**, 2064–2067 (2017).

(6) Blake, R. L., Hessevick, R. E., Zoltai, T. & Finger, L. W. Refinement of the hematite structure. *Am. Mineral.* **51**, 123–129 (1966).

(7) Tucker, M. G., Keen, D. A., Dove, M. T., Goodwin, A. L. & Hui, Q. RMCProfile: Reverse Monte Carlo for polycrystalline materials. *J. Phys.: Condens. Matter* **19**, 335218 (2007).

(8) McGreevy, R. L. & Pusztai, L. Reverse Monte Carlo simulation: A new technique for the determination of disordered structures. *Mol. Simul.* **1**, 359–367 (1988).

(9) Cumby, J. & Attfield, J. P. Ellipsoidal analysis of coordination polyhedra. *Nat. Commun.* **8**, 14235 (2017).
